# Genome Sequence of “*Candidatus* Carsonella ruddii” Strain BT from the Psyllid *Bactericera trigonica*

**DOI:** 10.1128/genomeA.01466-17

**Published:** 2018-01-25

**Authors:** Leron Katsir, Ruan Zhepu, Alon Piasezky, Jiandong Jiang, Noa Sela, Shiri Freilich, Ofir Bahar

**Affiliations:** a Department of Plant Pathology and Weed Research, Agricultural Research Organization, Volcani Center, Rishon LeZion, Israel; b Newe Ya’ar Research Center, Agricultural Research Organization, Ramat Yishay, Israel; c Department of Microbiology, College of Life Sciences, Nanjing Agricultural University, Nanjing, China; d The Robert H. Smith Faculty of Agriculture, Food and Environment, the Hebrew University of Jerusalem, Rehovot, Israel

## Abstract

The genome of “*Candidatus* Carsonella ruddii” strain BT from *Bactericera trigonica* in Israel was sequenced. The full-length genome is 173,904 bp long and has a G+C content of 14.6%, with 224 predicted open reading frames (ORFs) and 30 RNAs.

## GENOME ANNOUNCEMENT

The intracellular gammaproteobacterium “*Candidatus* Carsonella ruddii” is an obligate symbiont of psyllids (Psylloidea, Hemiptera), which are plant phloem-feeding insects (1). “*Ca*. Carsonella ruddii” possesses one of the smallest bacterial genomes known and resides in specialized cells, bacteriocytes, where it is believed to synthesize amino acids missing from the psyllid’s diet (2).

Certain psyllid species are vectors of the plant-pathogenic “*Candidatus* Liberibacter” species bacteria. The carrot psyllid *Bactericera trigonica* and potato psyllid *Bactericera cockerelli* are vectors of “*Ca*. Liberibacter solanacearum,” the associated pathogen of carrot yellows and potato zebra-chip diseases, respectively (3–5). Another psyllid vector, *Diaphorina citri*, transmits “*Ca*. Liberibacter asiaticus,” the causal agent of huanglongbing (citrus greening disease) (6). We report here the sequence of “*Ca*. Carsonella ruddii” associated with the carrot psyllid *B. trigonica* from Israel.

From an established colony of field-collected *B. trigonica*, adult psyllids were harvested and flash frozen. Genomic DNA was prepared from three samples, one containing 4 adult psyllids (7) and the other two containing 6 psyllid nymphs each (8). The nymph samples underwent DNA amplification using the Qiagen REPLI-g minikit. One of the nymph samples was further purified using the QIAamp DNA minikit. Whole-genome sequencing was performed with an Illumina Miseq instrument (150 bp, paired end) at the Technion Genome Center in Haifa. Generated sequences were cleaned from low-quality sequences and subjected to adapter removal using Trimmomatic v 0.32 (9). The library was assembled using an A5-miseq assembler (10). A total of 30,268,912 reads were pooled from all three samples, 109,441 (~0.36%) mapped to “*Ca*. Carsonella ruddii.” A BLAST search of assembled contigs found the complete “*Ca*. Carsonella ruddii” genome on one contig. The genome was submitted to the Joint Genome Institute (JGI) IMG/M system (11) for annotation.

The genome was found to be 173,904 bp, with a G+C content of 14.6%, containing 224 genes (194 protein-coding genes) and 30 RNAs (1 16S rRNA, 1 23S rRNA, and 28 tRNAs). We compared the average nucleotide identity (ANI) of “*Ca*. Carsonella ruddii” from *B. trigonica* (“*Ca*. Carsonella ruddii” strain BT) reported here with the ANIs of the following nine “*Ca*. Carsonella ruddii” genome assemblies listed in NCBI: *Pachypsylla venusta* (strain PV) (GenBank assembly accession number GCA_000010365) (12), *Ctenarytaina eucalypti* (strain CE) (GCA_000287235) (13), *Ctenarytaina spatulata* (strain CS) (GCA_000287255) (13), *Heteropsylla cubana* (strain HC) (GCA_000287275) (13), *Heteropsylla texana* (strain HT) (GCA_000287295) (13), *Pachypsylla* sp. “celtidis” (strain PC) (GCA_000287315) (13), *Diaphorina citri* (strain DC) (GCA_000441575) (1), *D. citri* (strain YCCR) (GCA_001274515) (14), and *B. cockerelli* (strain BC) (GCA_002009355) (15). We found that “*Ca*. Carsonella ruddii” strain BT has the strongest relationship by ANI to “*Ca*. Carsonella ruddii” strain BC (89.9%), which is the primary endosymbiont of the same psyllid genus (*Bactericera*). The lowest ANI score is with “*Ca*. Carsonella ruddii” strain PC (73.89%). Between “*Ca*. Carsonella ruddii” strains BT and DC, the ANI was 79.38%. The low ANI values shared between “*Ca*. Carsonella ruddii” strains may be the result of limiting replicative environments, as well as the effect that the loss of DNA repair enzymes has on genome evolution (2). The genome sequence of “*Ca*. Carsonella ruddii” strain BT will aid in understanding the geographic, host, and microbial relationships that drive “*Ca*. Carsonella ruddii” evolution.

### Accession number(s).

The complete genome sequence of “*Ca*. Carsonella ruddii” strain BT is deposited at GenBank, NCBI, under the accession number CP024798.
